# Lung Cancer Presenting as Upper Extremity Musculoskeletal Pain: A Case Report

**DOI:** 10.7759/cureus.28706

**Published:** 2022-09-02

**Authors:** Ben Weber, Nicholas D Luke, Alyssa M Payette, Hamid Shaaban

**Affiliations:** 1 Hematology and Oncology, St. Michael Medical Center, Newark, USA; 2 Medical School, St. George's University School of Medicine, True Blue, GRD

**Keywords:** smoker, metastasis, critical care, musculoskeletal injury, adenocarcinoma

## Abstract

Adenocarcinoma is a tragically common iteration of lung cancer. Risk factors included primary or secondary exposure to tobacco smoke, family history of the disease, and occupationally related hazards, among others. Metastasis to various distant organs may present quite late and in unusual ways, providing a challenge to healthcare providers. A combination of imaging, biopsy, and histochemical analysis can be used to clinch the diagnosis and guide management. Effective treatment relies on a prompt diagnosis, from surgery to radiation and chemotherapy. Our case illustrates how an advanced metastatic lung cancer clinically manifested as something as seemingly benign as shoulder pain.

## Introduction

Adenocarcinoma is the single most common form of lung cancer known. It accounts for roughly 40-50% of lung cancer diagnoses and tends to involve the periphery of the lung [1]. There is a strong correlation with a history of smoking, but it continues to be the predominant subtype for non-smokers [1]. Driver mutations have been implicated in developing adenocarcinoma-related tumors, including epidermal growth factor receptor (EGFR) and anaplastic lymphoma kinase (ALK) [2]. Immunohistopathologic analysis from biopsy samples can provide insights into cell origin and guide treatment targets. According to US Preventive Services Task Force (USPSTF) recommendations, guideline-directed screening can help detect problematic nodules early and improve outcomes [1]. Lung cancer metastasizes to the central nervous system, the liver, the axial skeleton, the adrenals, and the liver. So, the treatment approach heavily depends on the stage at the time of presentation. We report a patient diagnosed with lung adenocarcinoma whose initial manifested as subacute, worsening musculoskeletal pain with no other associated symptoms.

## Case presentation

Our patient is a 76-year-old female who was a previous smoker with a 5-pack year history, and with a past medical history of heart failure with preserved ejection fraction, atrial fibrillation (managed with rate control via metoprolol 100 mg, and apixaban 5 mg for anticoagulation) and Wolf-Parkinson-White Syndrome status post ablation, coronary artery disease with five stents, pacemaker, previous myocardial infarction, neuropathy, and seizure disorder who presented to the emergency department (ED) with shoulder pain. The pain started several weeks before the presentation with left-sided shoulder pain with radiation down the ventral aspect of her arm down to the elbow. Soon after, she began to experience progressive right-sided shoulder pain with a similar pattern of radiation to the elbow. She denied any recent trauma, history of exposure to significant radiation, and a history of malignancy. She first presented to an urgent care for this complaint, received chest radiography, and was then told she had fractures in the bilateral seventh ribs, left posterior second rib, left posterior sixth rib, and right posterolateral ninth rib, despite no recent trauma. She denied any other active complaints at the time of admission. On presentation, vital signs were 98.1 degrees Fahrenheit, blood pressure was 121/68 mmHg, heart rate was 84 beats per minute, respiratory rate was 16 per minute, and oxygen saturation was 97% on room air. She was awake, alert, oriented, and in no acute respiratory distress. Moderate bilateral crackles and lower extremity edema (+1) were appreciated, while neurologic and abdominal exams were grossly within normal limits. 

**Figure 1:**
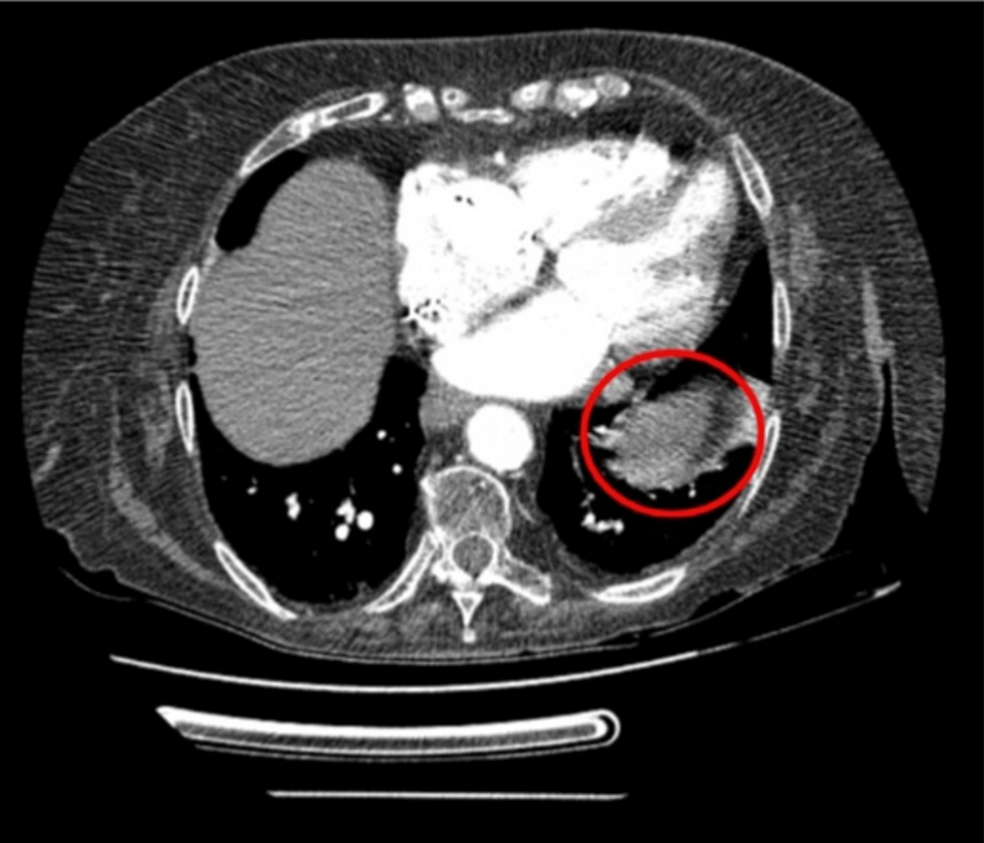
Computed tomography with angiography depicting an amorphous area of mass-like consolidation in the anterior left lower lobe at the left lung base (red circle).

**Figure 2:**
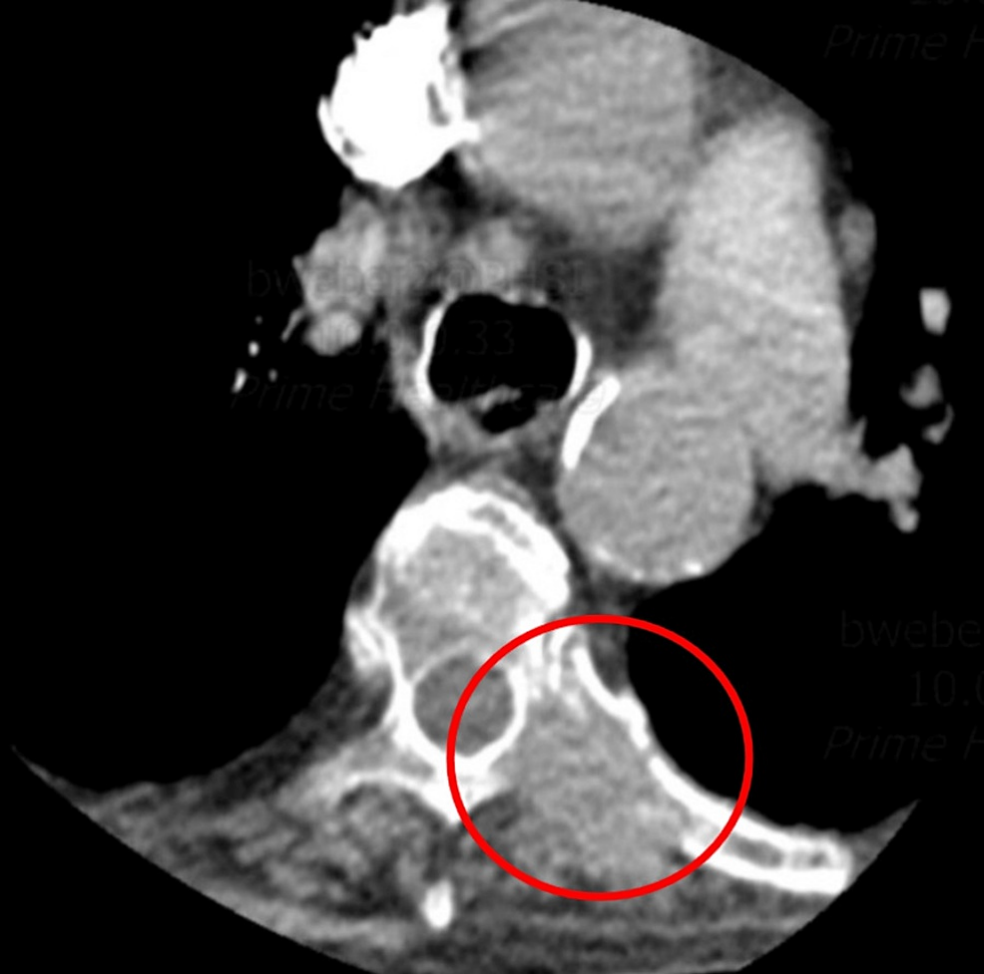
Computed tomography of the thoracic spine depicting possible osseous metastases to the ribs (red circle).

Significant laboratory values included brain natriuretic peptide (BNP)of 408.51 pg/mL (normal < 100 pg/mL), high sensitivity troponin I of 52 ng/mL (normal: 0 - 0.04 ng/mL), alkaline phosphatase 197 U/L (normal: 44 - 147 U/L), and D-Dimer of 590 mg/L fibrinogen equivalent units​​​​​​​ (FEU)(normal < 0.5 mg/L). A computed tomographic angiogram of the chest with contrast showed an area of mass-like consolidation in the left lower lobe (Figure 1). Scattered destructive osseous lesions involving the spine and bilateral ribs were noted as well. Computed tomography also noted an epidural extension of the tumor along the left aspect of the thecal sac at T3 resulting in spinal canal stenosis with potential for cord compression. A four-centimeter left adrenal mass was noted. CT cervical spine showed vertebral lytic lesions at C5, T1, T2, and T3, and epidural mass at T3 with impending spinal cord compression. Computed tomography of the thoracic spine also depicted possible osseous metastases to the bilateral ribs (Figure 2) with a chronic compression fracture of T12 with severe loss of height anteriorly (not shown). CT lumbar spine showed multiple lumbosacral and bilateral iliac lytic lesions. Since there was no evidence of metastasis to the shoulder joints, the patient's pain was determined to be referred pain due to direct extension of the tumor into the thecal sac at the level of T3. 

The patient was started on oral prednisone (60 mg) daily in case of possible cord compression. Oral prednisone was transitioned to intravenous decadron (8 mg) every eight hours and then tapered off once cord compression was ruled out via a spinal myelogram. A bone biopsy of the iliac crest confirmed the diagnosis of metastatic adenocarcinoma of the lung. Over two weeks, the patient was started on a regimen of 3000 cGy radiation in 10 fractions of 300 cGY. The patient was kept in a cervicothoracic brace for stabilization. Immunohistochemistry, PDL1 (programmed death-ligand 1) testing, and NGS (Next Generation Sequencing) were sent. 

Eventually, her condition deteriorated with a new onset of septic shock and reoccurrence of atrial fibrillation (her atrial fibrillation was treated with ablation prior to this new event of sepsis) with a rapid ventricular response. She was stabilized on intravenous cefepime and doxycycline for suspected pneumonia, amiodarone for rate control, vasopressin, and levophed for blood pressure support. In light of an acute episode of a bloody bowel movement, an endoscopy was performed, which was unremarkable, and a colonoscopy was planned but aborted secondary to patient refusal. Her medical condition was deemed stable for management on the medical floor. However, the patient ultimately deteriorated before transitioning to hospice on day 25.

## Discussion

Lung adenocarcinoma is the most common primary lung cancer in the United States. It accounts for 40% of all lung cancers. One longitudinal study of non-small cell lung cancer by Kocher indicates that the most common presenting symptoms tend to be cough (54.7%) and dyspnea (45.3%) and, unfortunately, are most frequently diagnosed in stage IV (37.9%) [3]. Our patient’s sole complaint was chronic, progressive shoulder pain. Other cases may present as neurovascular mass effects, including horner syndrome, superior vena cava obstruction, and brachial plexus compression. In some cases, paraneoplastic syndromes have also been observed: Lambert-Eaton and Cushing syndromes, hypertrophic osteoarthropathy, etc [1]. Musculoskeletal pain is so ubiquitous that it may be disregarded as merely age-related degenerative changes. When chronic and progressive, this should prompt a clinical re-evaluation of the situation. CT chest can be used to screen and, if positive, can lead down an algorithm to rule out further organ involvement - CT thorax and abdomen, brain MRI, bone scan, PET scan, and bronchoscopy [1]. Genomics can be utilized by clinicians as well to dictate the path of treatment and management [2]. Biomarkers such as EGFR and KRAS mutations and ALK rearrangements can focus the treatment by targeting these oncogenic alterations rather than applying standardized chemotherapy to all patients [2,4]. Our patient underwent multiple testing after initial imaging demonstrated adrenal, rib, and axial spine involvement. 

Even with treatment, the survival rates of lung adenocarcinoma are somewhat bleak. In one study, the average life expectancy of patients with lung adenocarcinoma was 12.60 ± 1.59 months [5]. The same study estimated that the five-year relative survival rate was 8% (18% for women and 7% for men) [5]. Even with treatment, the gender of the patient and the presence of bone or liver metastasis (our patient had bone metastases) played a statistically significant role in patient survival rates and management [5, 6]. 

Our patient eventually passed away due to drastic progression of lung adenocarcinoma metastases. The advancement of cancer had gone unchecked due to delayed diagnosis and lack of symptomatology. Unfortunately, this late presentation is a common feature of lung adenocarcinoma, and it creates a challenging atmosphere for the possibility of surgical resection. Ellis and Vandermeer discussed how Canada had about 23,000 cases and 19,900 deaths from lung malignancies in 2007, and only 20-30% of those cases had an early-stage diagnosis with the potential of resection [7]. One possible cause of the delayed diagnosis is that lung malignancy may mimic other respiratory tract diseases and cloud the differential diagnosis. Ellis and Vandermeer also noted that the type of symptoms the patient presented with might influence the time to diagnosis [7]. If a patient presented with atypical symptoms, including bone or joint pain, the referral to a specialist took a median of 104 days. In contrast, typical symptoms (cough, dyspnea, hemoptysis) took a median of 29 days for a specialist referral [7]. 

The development of early detection systems is crucial for increasing the survival rates for lung malignancies; however, few reliable screening tests exist at the moment. Computed tomography of the chest without contrast may be used as a screening tool in those with a chronic smoking history (>20 years, along USPSTF guidelines); however, our patient had a 5-pack year smoking history and would not meet the criteria. Genomic testing may have the potential to fill the placeholder in the future. Until a solution is found, general practitioners should be wary of unexplained respiratory symptoms and send patients for specialist referrals as early as possible [8]. 

Advanced metastatic cancer can be challenging to manage, and the discussion of palliative care should be addressed as soon as possible. The addition of palliative care to a patient’s routine management with metastatic cancer emphasizes comfort and quality of life improvements. Ferrell et al mention the responsibility of clinicians to identify early and reversible causes of discomfort in patients and discuss ways to address them [9]. The discomfort may be the result of the primary tumor itself, and it may be due to the metastatic aspect of the cancer. It also can result as an adverse effect of the treatment or due to paraneoplastic syndromes [9, 10]. The delayed diagnosis in our patient amplified the discomfort that she had to endure, and this made it even more challenging to control.

## Conclusions

This case depicts an unusually isolated and mild chief complaint in the context of widely metastatic lung adenocarcinoma. A strictly clinical outpatient evaluation without advanced, high-resolution imaging would have been grossly insufficient to diagnose our patient’s underlying cancer. Highly atypical cases of malignancies may present insidiously, despite recent advances in medicine. Unfortunately, as in the case of our patient, it may elude even seasoned physicians until the prognosis becomes extremely poor.
